# Integrated analysis of transcriptome, sRNAome, and degradome involved in the drought-response of maize Zhengdan958

**DOI:** 10.1515/biol-2022-1044

**Published:** 2025-01-27

**Authors:** Shuqiong Yang, Jiafei Liu, Lingling Cao, Jibao Chen, Pengfei Duan

**Affiliations:** Henan Provincial Key Laboratory of Ecological Security for Water Source Region of Mid-line of South-to-North Diversion Project, Nanyang Normal University, Nanyang, 473061, China

**Keywords:** maize, drought stress, transciptome, sRNAome, degradome

## Abstract

Drought is a major abiotic stress in restricting the growth, development, and yield of maize. As a significant epigenetic regulator, small RNA also functions in connecting the transcriptional and post-transcriptional regulatory network. Further to help comprehending the molecular mechanisms underlying drought adaptability and tolerance of maize, an integrated multi-omics analysis of transcriptome, sRNAome, and degradome was performed on the seedling roots of an elite hybrid Zhengdan958 under drought stress. In this study, 2,911 genes, 32 conserved miRNAs, and 12 novel miRNAs showed a significantly differential expression under drought stress. Moreover, 6,340 target genes of 445 miRNAs were validated using degradome sequencing, forming 281 miRNA–mRNA pairs in control (CK) and drought-stressed (DS) library. These target genes were mainly involved in the plant hormone signal transduction and phenylpropanoid biosynthesis pathways. The integrated multi-omics analysis revealed that five DEmiRNA–mRNA pairs displayed negatively correlated expression patterns, which were also verified by qRT-PCR. Tissue-specific expression profile and regulatory network analysis revealed that miR528a/b-*Zm00001d021850*, miR408a/b-*Zm00001d020794*, and miR164e-*Zm00001d003414* might be essential in root-specific drought stress response of maize Zhengdan958 seedlings. These worthwhile will promote the functional characterization of miRNA–mRNA modules response to drought stress, and potentially contribute to drought-resistance breeding of maize.

## 1 Introduction

On a global scale, drought stress is the recurring environmental threat to negatively influence crop survival and productivity. When combined with global warming, drought stress is likely to display a crucial effect to the environment, which also leads to other abiotic stresses [1]. The physiological acclimation of single plant may buffer the influence of drought; hence, it is vital to further improve the drought adaptation of plants [2].

While plants are sessile organisms, they have sophisticated drought stress-responsive mechanisms involving numerous genes that protect them against the environmental harms [3]. The gene expression could be regulated at the transcriptional, post-transcriptional, and epigenetic modification levels [2]. As the endogenous noncoding RNAs, small RNAs (21–26 nt) mainly including small interfering RNAs (siRNAs) and microRNAs (miRNAs), function as sequence-specific regulators in varied biological processes at the transcriptional and post-transcriptional levels [2,4]. MiRNAs are important regulators, ubiquitously function at the post-transcriptional expression regulation of their target genes via transcript cleavage or translational inhibition, which play key roles in diverse aspects of plant growth and development along with abiotic and biotic stress responses [5]. In plants, the conceivable regulation patterns of drought-responsive miRNAs have been reported in *Arabidopsis thaliana*, *Triticum aestivum* L., *Saccharum officinarum*, *Oryza sativa* L., and *Sorghum bicolor* [6–9]. The expression patterns of miR156, miR166, miR169, and miR396 were conserved in multiple plants, which were up (or down)-regulated expression under high salt or drought stress [10]. In the transgenic lucerne (*Medicago sativa* L.) plants, the overexpression of miR156 not only improved the drought tolerance of transgenic plants but also enhanced the abscisic acid (ABA), proline, and antioxidant levels [11]. The knockdown of miR166 altered the stem xylem development and caused the leaf rolling of the knockdown rice variety Nipponbare (*O. sativa* L. ssp. japonica) lines, conferring the miR166 knockdown lines with higher drought resistance [12].

To better understand the drought-responsive functions of plant miRNAs, it is crucial to identify their target genes as well as the expression patterns of target genes. Degradome sequencing is a high-throughput technique base on parallel analysis of RNA ends, which has successfully been used to identify new miRNAs and their target genes, assess miRNA self-regulation, and characterize the relationship between miRNA and their target genes [13]. Degradome sequencing has enabled the identification of miRNAs and target genes related to plant growth and development, biotic and abiotic stress responses, and terpenoid biosynthesis in maize, *A. thaliana*, *Populus*, and *Hemerocallis fulva* [14,15]. A water-deficit regulated maize transcript, similar to known miR399 target mimics, was identified and hypothesized as another regulatory player, moderating the role of miR399e, miR399i, and miR399j-3p in the primary root growth zone water deficit responses [16]. Through affecting SPLs (SQUAMOSA promoter binding protein like), the flavonoid metabolism, and the lignin synthesis of anther walls pathway, the miR156, miR5488, and miR399 affect the male fertility of PA64S in *O. sativa* [17]. When subjected to different stresses, the integrated multi-omics analysis of durum wheat identified the key miRNA–mRNA pairs with opposed regulatory patterns [18].

Maize (*Zea mays* L.) is one of the main feed crop and grain products around the world, which is also widely applied as a model crop of genetics research. Owing to the high water requirements as well as long growing season, maize is also particularly sensitive to drought stress [19]. Through utilizing different genetic and breeding approaches, efforts are in progress to improve the drought tolerance of maize [20]. The drought tolerance of maize could be considerably improved by combining multi-omics technologies with novel breeding methods and high-throughput phenotyping [21]. Though the water requirement of maize at the seedling stage is below the other development stages, drought also affected the seedling adaptability to moisture levels along with potential yield reduction of maize [22]. Hence, elucidating the drought-responsive mechanism of seedlings is vital to improve the drought tolerance of maize. Zhengdan958 is a mid- and late-maturing hybrid with high planting density, good stress-tolerance, and high but stable yield, which has become one of the most widely planted maize cultivars in Huang-Huai-Hai plain of China in the past 20 years. In this study, we integrated transcriptome, sRNAome, and degradome using multi-mics to identify drought-adaptive miRNA–mRNA pairs in the seedling roots of Zhengdan958 after PEG-6000 treatment. Furthermore, we aimed to reveal potential regulation patterns of drought-adaptive miRNA–mRNA pairs. This study will provide valuable gene-level fundament of the drought-responsive networks in maize, and help to improve the genetic engineering of drought-tolerant maize.

## 2 Materials and methods

### 2.1 Plant materials and drought stress treatment

Seeds of the maize elite hybrid Zhengdan958 (market purchase) were surface-sterilized using 75% ethanol for 5 min, rinsed with ddH_2_O three times, and then germinated for 48 h in a wet rolled brown paper towel at 28°C. After germination, 4-day-old seedlings were subjected to drought treatment till Day 8 using Hoagland’s solution with or without 25% (w/v) PEG-6000. The seedlings were cultured under a controlled condition with a 16 h photoperiod (8:00–24:00), 300 µmol m^−2^ s^−1^ photons, 28/23°C (day/night), and 30–50% relative humidity. Three biological replicates of control (CK) and drought-stressed (DS) underground roots were sampled at Day 8 with 15 plants per treatment, and then immediately stored using liquid nitrogen.

### 2.2 Morphology measurement and data analysis

Determination of growth index: take out the seedlings from the pot, rinse the vermiculite attached to the roots with ddH_2_O, and suck the excess water using the filter paper before measuring the indicators. When measuring the indexes, the root length (RL), root surf area (RS), root avg diam (RA), and root volume (RV) were scanned using a Epson flat-bed scanner and analyzed with WinRHIZO, and then weighed the root fresh weight (RFW), shoot fresh weight (SFW), root dry weight (RDW), shoot dry weight (SDW), total fresh weight (TFW), and total dry weight (TDW) with an electronic analytical balance. All the morphological data of maize seedling roots were processed using SPSS 25.0, followed by a two-way analysis of variance (ANOVA) and compared at *p* < 0.05 and 0.01.

### 2.3 RNA extraction, library construction, and sequencing

The TRIzol reagent (Invitrogen, Carlsbad, USA) was used to extract total RNA from each root sample of control and drought-treated Zhengdan958 seedlings. NanoDrop One (NanoDrop, Wilmington, USA) and Agilent Bioanalyzer 2100 (Agilent, Santa Clara, USA) were used to analyze the quantity and purity of total RNA (RIN > 7.0). For preparing transcriptome sequencing library, approximately 15 µg of root RNAs were used for each biological sample of Zhengdan958 seedling. Paired-end transcriptome sequencing was performed by Biomarker Technologies Corporation, Beijing, China.

Based on the protocol of NEB Next Ultra small RNA Sample Library Prep Kit (New England Biolabs, Ipswich, USA), approximately 6 µg of equally mixed RNAs was prepared from control and drought-treated root samples of Zhengdan958 seedlings. A degradome library including approximately 20 μg of equally mixed RNAs was constructed from six Zhengdan958 root samples. Both small RNAome and degradome sequencing were performed using Illumina HiSeq 2500 platform according to manufacturer-recommended protocol (Biomarker Technologies Corporation, Beijing, China).

### 2.4 Transcriptome data analysis

The adaptors, empty, low quality, and one-copy tags were filtered from the raw data to obtain clean reads. Clean reads then were aligned to the Zm-B73-REFERENCE-NAM-5.0 (https://www.maizegdb.org/) using the HISAT2 program (http://ccb.jhu.edu/software/hisat2/index.shtml). *De novo* assembly of the transcriptome was performed using StringTie (CCB of Johns Hopkins University, Baltimore, USA). FeatureCounts (WEHI, Parkville Victoria, Australia) was used to count the read numbers mapped to each gene. The number of tags corresponding to each gene was calculated and normalized to fragments per kilobase per million (FPKM) reads to analyze the gene expression. Genes with *p* ≤ 0.001 were considered to have altered their expression and designated as the differentially expressed gene (DEG) using the DESeq2 package in R. GO annotation of DEGs was implemented using GOseq R packages with the Wallenius non-central hyper-geometric distribution model, and KEGG Automatic Annotation Server provided functional annotation of genes by blasting against the manually managed KEGG database [23]. The statistical enrichment of DEGs in KEGG pathways was tested using KOBAS-i [24].

### 2.5 MiRNA data analysis

First, in-house perl scripts were used to process the raw reads. Then clean reads were obtained by filtering adapters, low-quality reads, repeats, and common RNA families (rRNA, tRNA, snRNA, snoRNA, and other ncRNA). Filtered unique sequences (18–30 bp) were used for detecting known and novel miRNA by blasting against the precursors of specific species from miRBase (http://www.mirbase.org/). There were two categories of known miRNAs, one category included unique sequences that match with one of the arms in the stem-loops of species-specific mature miRNAs. Any unique sequences that mapped to the other arm in the stem-loops of known species-specific precursors opposite to the annotated mature miRNA-containing arm belong to the other category. Un-mapped sequences were aligned against the specific genomes, and the hairpin RNA structures containing sequences were predicated from the flanking 120 nucleotides using RNAfold software (http://rna.tbi.univie.ac.at/cgi-bin/RNAWebSuite/RNAfold.cgi). These miRNAs with at least twofold change in expression (|log2(FC)| ≥ 1 and FDR ≤ 0.01) were identified here as differentially expressed miRNAs (DEmiRNAs). The expression patterns of DEmiRNAs were measured by systematic cluster analysis to explore the similarities and compare the relationships between the different libraries.

### 2.6 Degradome data analysis

After removing the linker sequences and low-quality sequences, the original tags were filtered out to obtain clean and cluster tags. Cluster tags were compared to the reference genome to get the distribution of tags. After comparing cluster tags with the Rfam database (https://rfam.org/), non-coding RNAs were identified and annotated, while the rest of the unannotated sequences were used for subsequent degradation position analysis. Information from the miRNA database and the sequence of predicted miRNAs were used to detect the degradation site by Clearland 3.0 [25], with the condition *p* < 0.05. The target genes were compared with NR, Swiss-Prot, GO, KEGG, and COG databases by BLAST to obtain the annotation information of target genes.

### 2.7 Expression profiles of DEmiRNA–mRNA pairs

The PrimeScript^TM^ RT reagent kit with gDNA Eraser (Takara Bio-medical Technology Co., Ltd, Beijing) was used to synthesize the first strand of cDNA, with 1 µg of total RNA in each 20 µL reverse transcription mixture. Relative quantitative analysis was performed using the BIO-RAD iQ5 Optical Module under the following conditions: 95°C/30 s (1 cycle), 95°C/5 s, 60°C/20 s, and 72°C/20 s (40 cycles). Melting curves were performed on the PCR products to test if only one single product was amplified without primer-dimers and other bands. Three biological replicates of each sample were carried out in the presence of SYBR^®^ Premix Ex Taq^TM^ II (Takara Biomedical Technology Co., Ltd, Beijing), and the average value of each was used for analysis. A total volume of 20 µL reaction contained 10 µL SYBR^®^ Premix Ex Taq^TM^ II (2×), 1 µM each primer, and 2 µL cDNA. In the qRT-PCR reactions, *Zea-Actin* served as the internal control to normalize the data across samples and the 2^−ΔΔCt^ method was used to calculate the relative expression amounts. Statistical analysis of relative gene expression was carried out using SPSS 25.0, and the analysis of ANOVA was conducted using Duncan’s multiple range test with significant differences at *p* < 0.05 and 0.01.

The RNA-seq data from MaizeGDB (https://www.maizegdb.org/) were used for predicting the expression profiles of three DEmiRNA target genes in different tissues (internode, meristem, ear_primordium, embryo, endosperm, germinatin_kernel, pericarp/aleurone, leaf, pollen, female_spikelet, and silk). The expression profiles, as FPKM reads of all three DEmiRNA target genes were obtained. A tissue-specific expression histogram of three DEmiRNA target genes was drawn using the TBtools software with Euclidean distances.

## 3 Results

### 3.1 Morphology changes of Zhengdan958 seedlings

**Figure 1:**
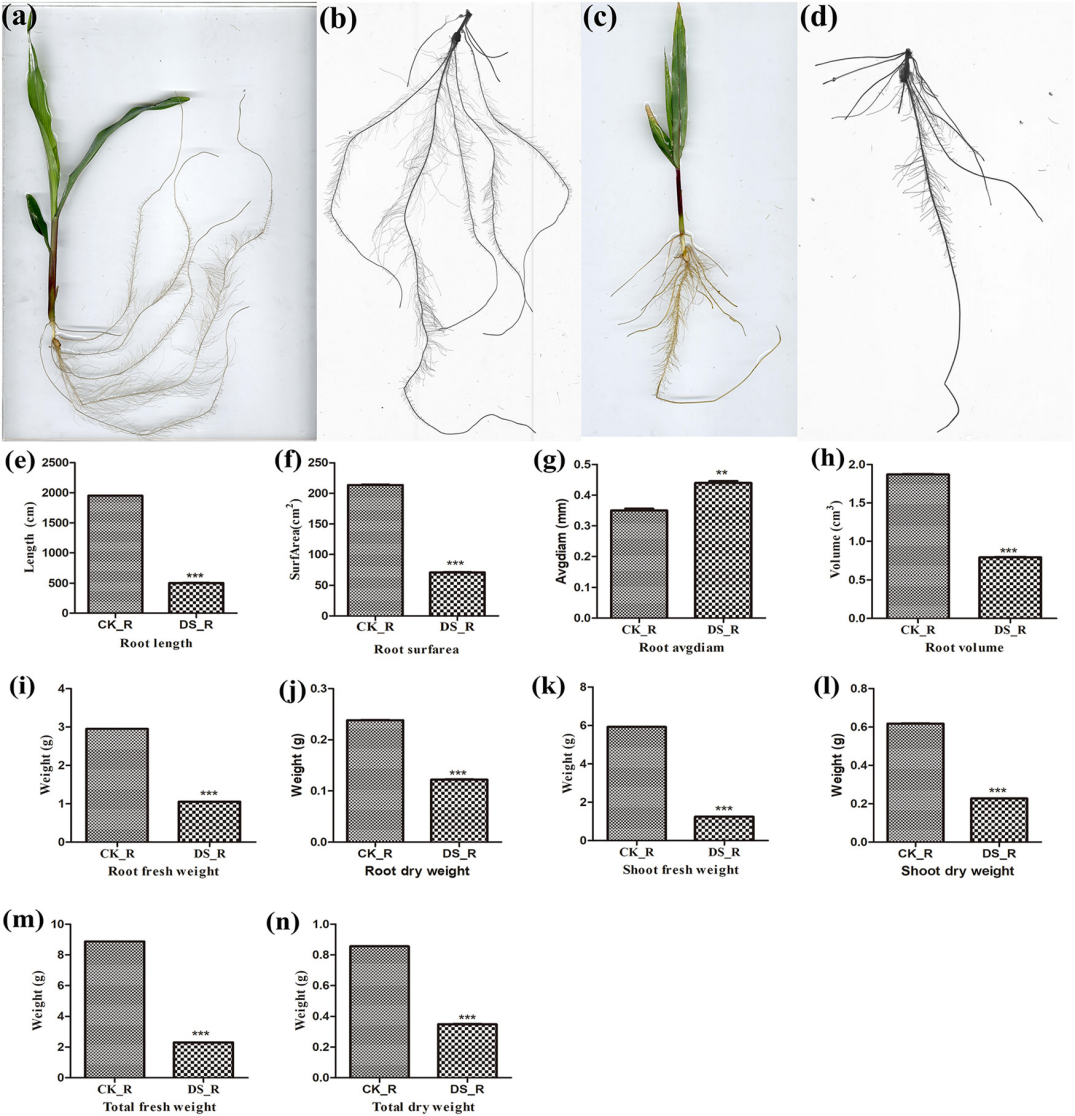
Morphological responses of Zhengdan958 seedlings to CK and DS treatments. (a, b) Phenotypes for Zhengdan985 seedlings under CK treatment. (c, d) Phenotypes for Zhengdan985 seedlings under DS treatment. The root length (e), root surfarea (f), root avgdiam (g), root volume (h), root fresh weight (i), root dry weight (j), shoot fresh weight (k), shoot dry weight (l), total fresh weight (m), and total dry weight (n) of Zhengdan958 seedlings under CK and DS treatments.

Compared with CK seedlings, DS-treated seedlings showed severe stressed phenotypes, with RL, RS, RV, RFW, RDW, SFW, SDW, TFW, and TDW showing significant differences (*p* < 0.01), which extremely decreased by 74, 67, 58, 64, 49, 79, 63, 74, and 59%, respectively (Figure 1). Compared with DS-treated seedlings, the RA of CK seedlings showed significant differences (*p* < 0.05), which increased by 26%.

### 3.2 Transcriptome sequencing of Zhengdan958 seedling roots under drought stress

**Figure 2:**
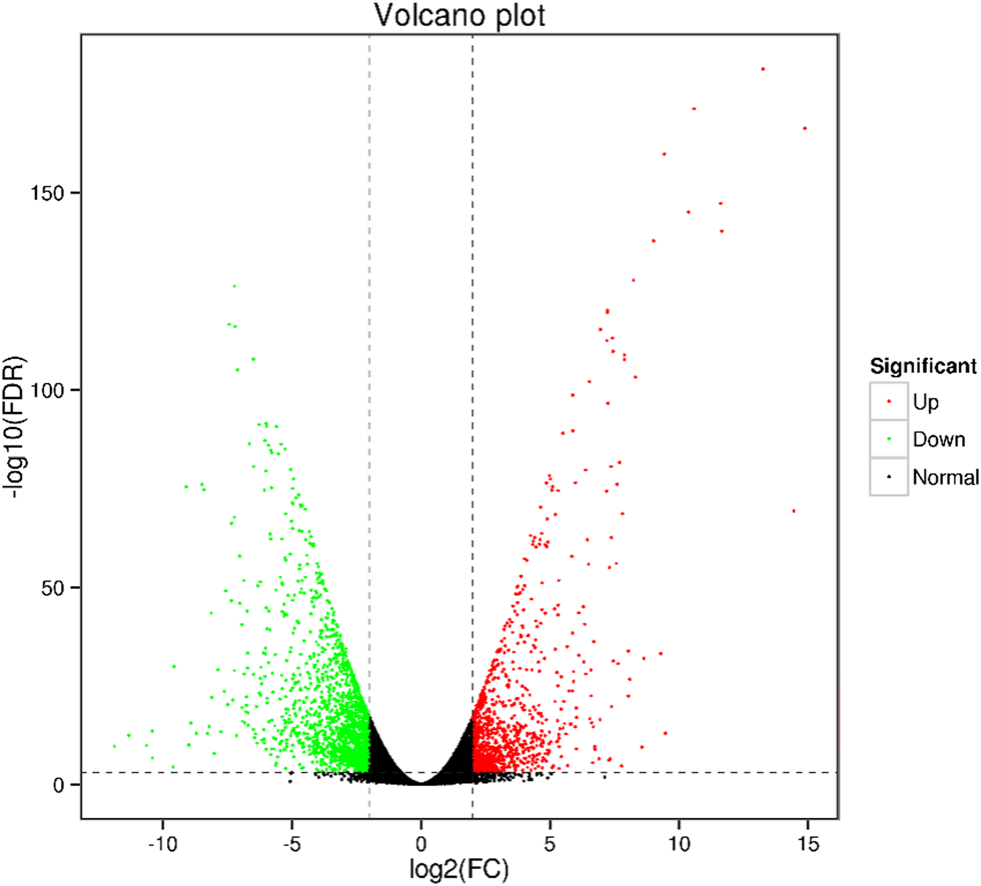
Volcano plot of DEGs from seedling roots after drought treatment.

Transcriptome sequencing of DS and CK root samples generated 43.32 GB clean data using Illumina Hiseq 2500; meanwhile, a total of 43,284 genes, 43,284 unique transcripts, 3,960 new genes, and 1,769,681 single nucleotide polymorphisms were identified separately. Of these, 1,515 GO annotated genes and 473 KEGG annotated genes were obtained separately. To identify drought-responsive DEGs, gene expression in CK was compared with that in DS treatment. A total of 2,911 DEGs were confirmed in drought-treated roots, of which 1,147 DEGs displayed an up-regulated expression while 1,764 DEGs showed a down-regulated expression (Figure 2).

**Figure 3:**
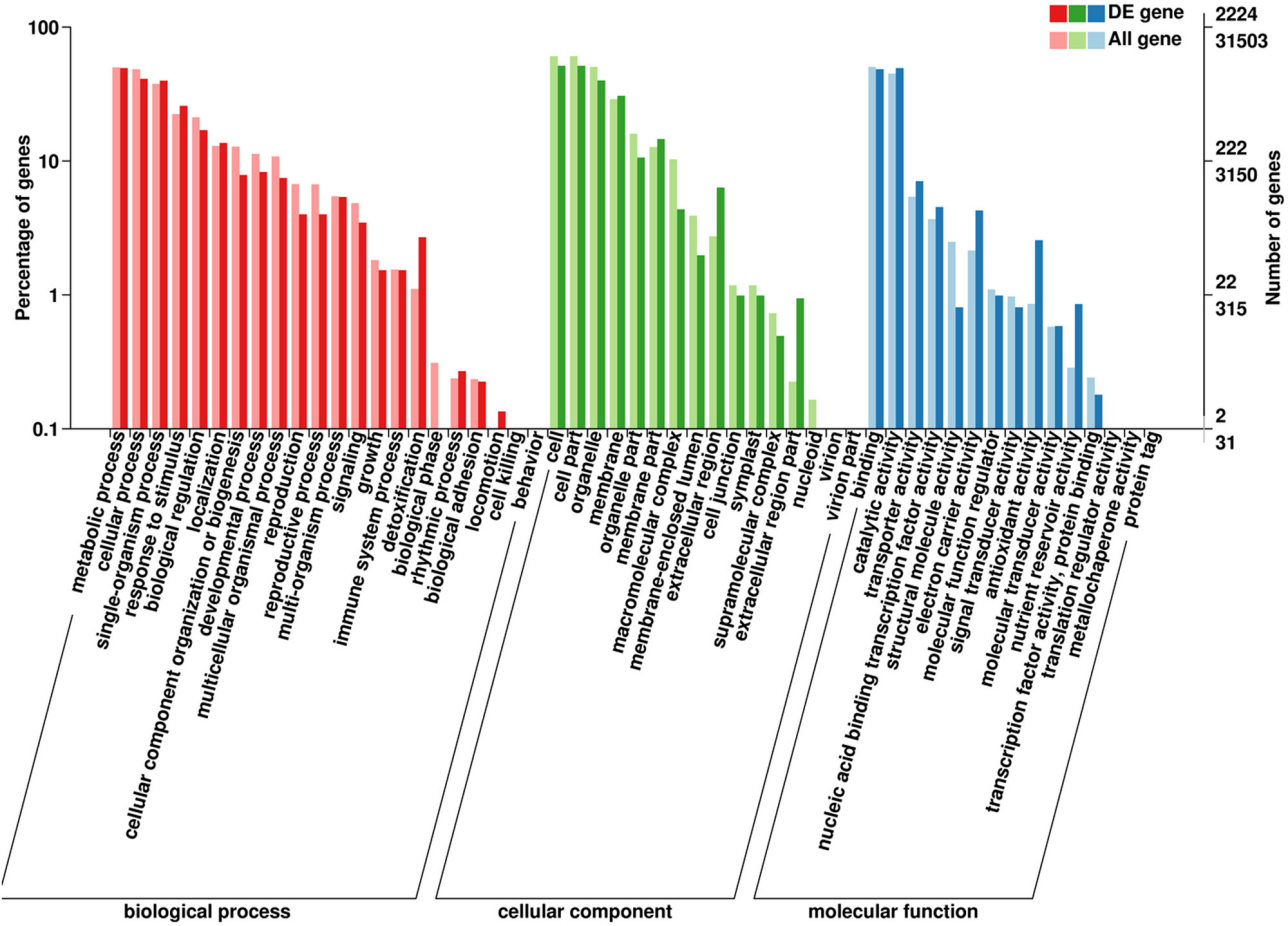
GO annotation for all DEGs.

GO annotation of 2,911 DEGs under drought stress was classified into three major categories, including molecular function, cellular components, and biological processes, having 1,178, 716, and 330 GO terms, respectively (Figure 3). These up-regulated 1,147 DEGs mainly enriched in DNA binding (GO:0003677), mitochondrion (GO:0005739), and protein serine/threonine kinase activity (GO:0004674), while the down-regulated 1,764 DEGs mainly enriched in mitochondrion (GO:0005739), cytoplasmic vesicle (GO:0016023), and DNA binding (GO:0003677).

**Figure 4:**
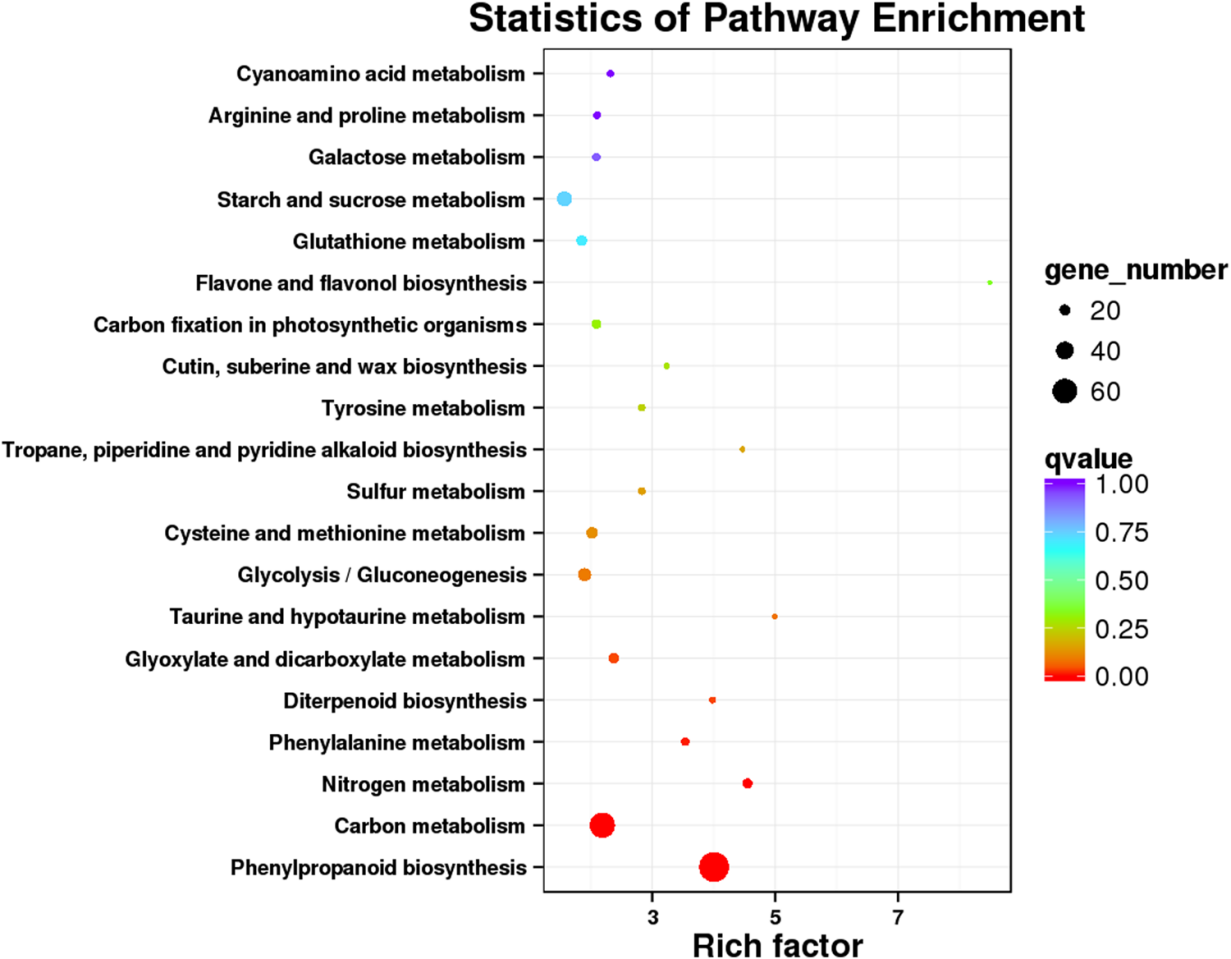
KEGG annotation for all DEGs.

For better understanding of the potential regulatory networks and gene function, the KEGG analysis of drought-responsive DEGs was performed. Top 20 enriched pathways were identified; the main enriched pathways were phenylpropanoid biosynthesis, carbon metabolism, and biosynthesis of amino acids (Figure 4). Compared with other pathways, the plant hormone signal transduction pathway was significantly enriched, indicating that the hormone might have played a vital role in the drought-responsive regulation of Zhengdan958 seedling roots.

### 3.3 SRNAome sequencing of Zhengdan958 seedling roots under drought stress

**Figure 5:**
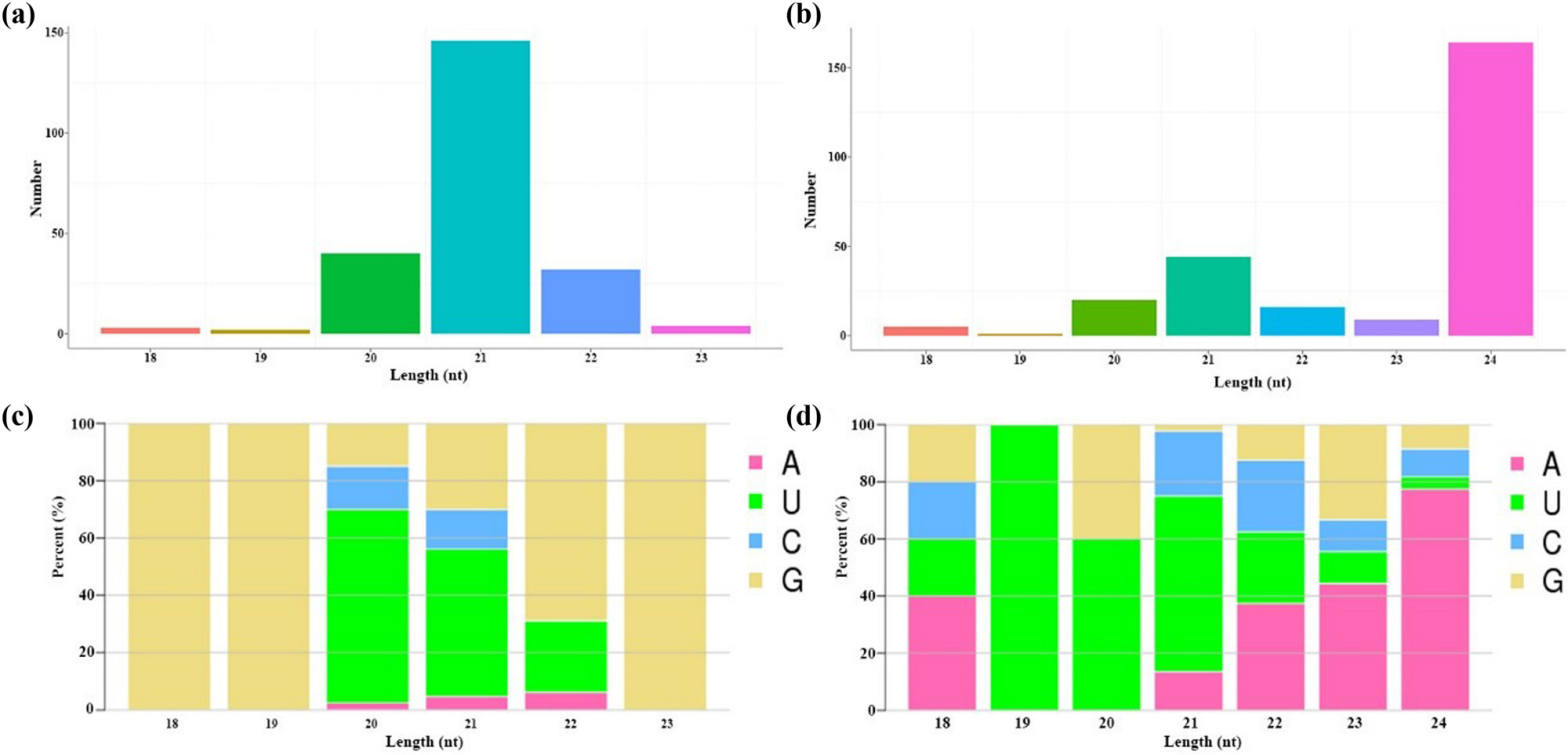
Size distribution and characterized analysis of maize drought-responsive miRNAs: (a) size distribution of known-miRNAs, (b) size distribution of novel-miRNAs, (c) first base preferences of mature known-miRNAs, and (d) first base preferences of mature novel-miRNAs.

Sequencing of small RNAs generated 124 million raw reads, about 86.4 million clean reads were obtained after removing these spliced and low-quality reads, containing snRNA, miRNA, snoRNA, tRNA, rRNA as well as unannotated reads. When compared with the reference genome, more than 28% of these above reads could be used to identify miRNAs. The 486 miRNAs of 18–24 nt in length were used for succeeding distribution analysis, including 227 known-miRNAs and 259 novel-miRNAs in the drought-responsive roots. Among these known-miRNAs, the most abundant was 21 nt miRNA, followed by 20 nt miRNA (Figure 5a). For a start, the first base of most mature 18–23 nt miRNAs was “G” (69–100%), while most 20 and 21 nt miRNAs used “U” (68 and 51%) (Figure 5c). Among these novel-miRNAs, 24 nt miRNA was the most abundant, followed by 21 nt miRNA (Figure 5b). Most mature 18–24 nt miRNAs used “A” as the start base (38–77%), while most 19, 20, and 21 nt miRNAs used “U” (60–100%) (Figure 5d).

**Figure 6:**
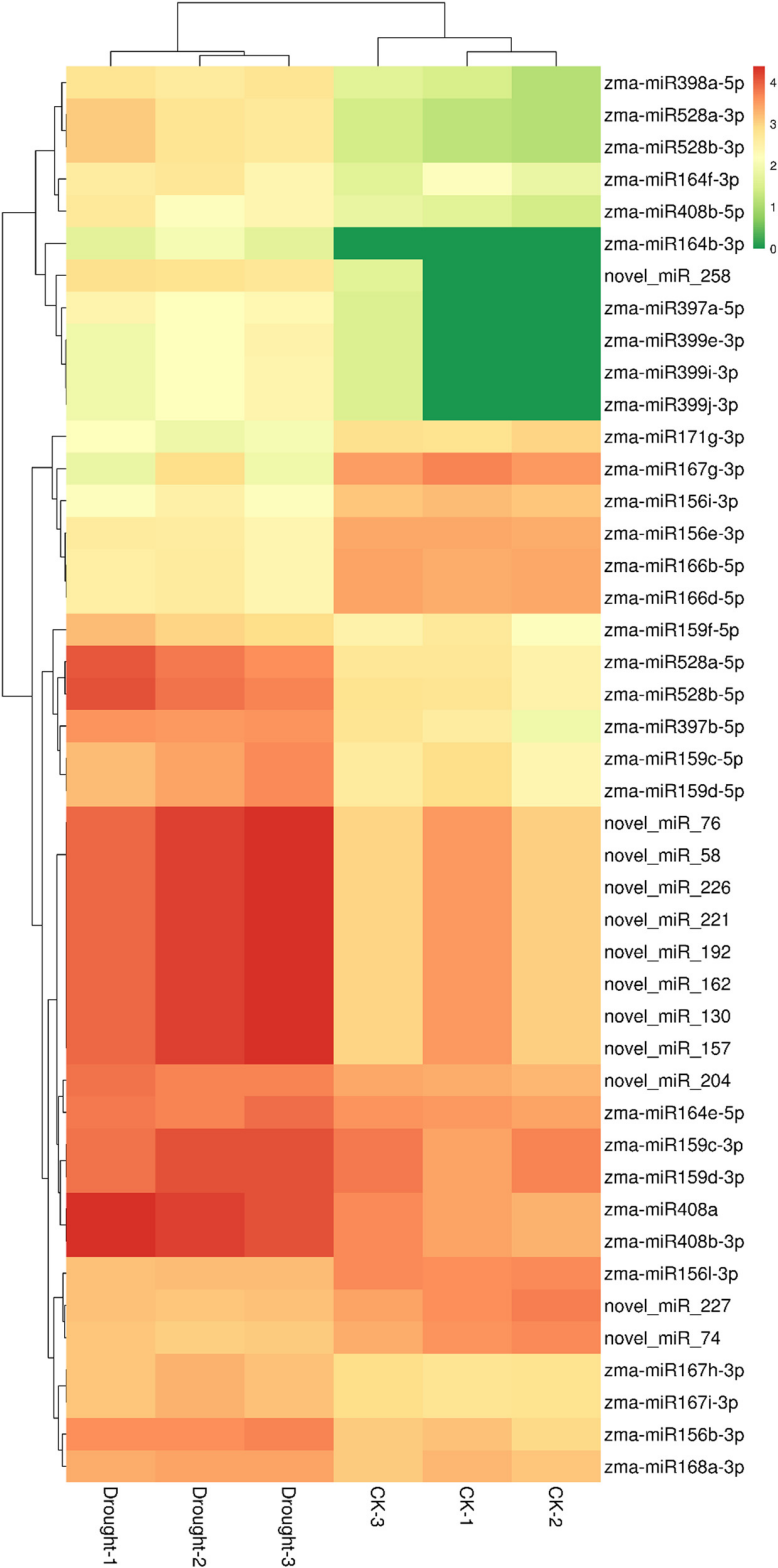
Expression heatmap of the DEmiRNAs.

**Figure 7:**
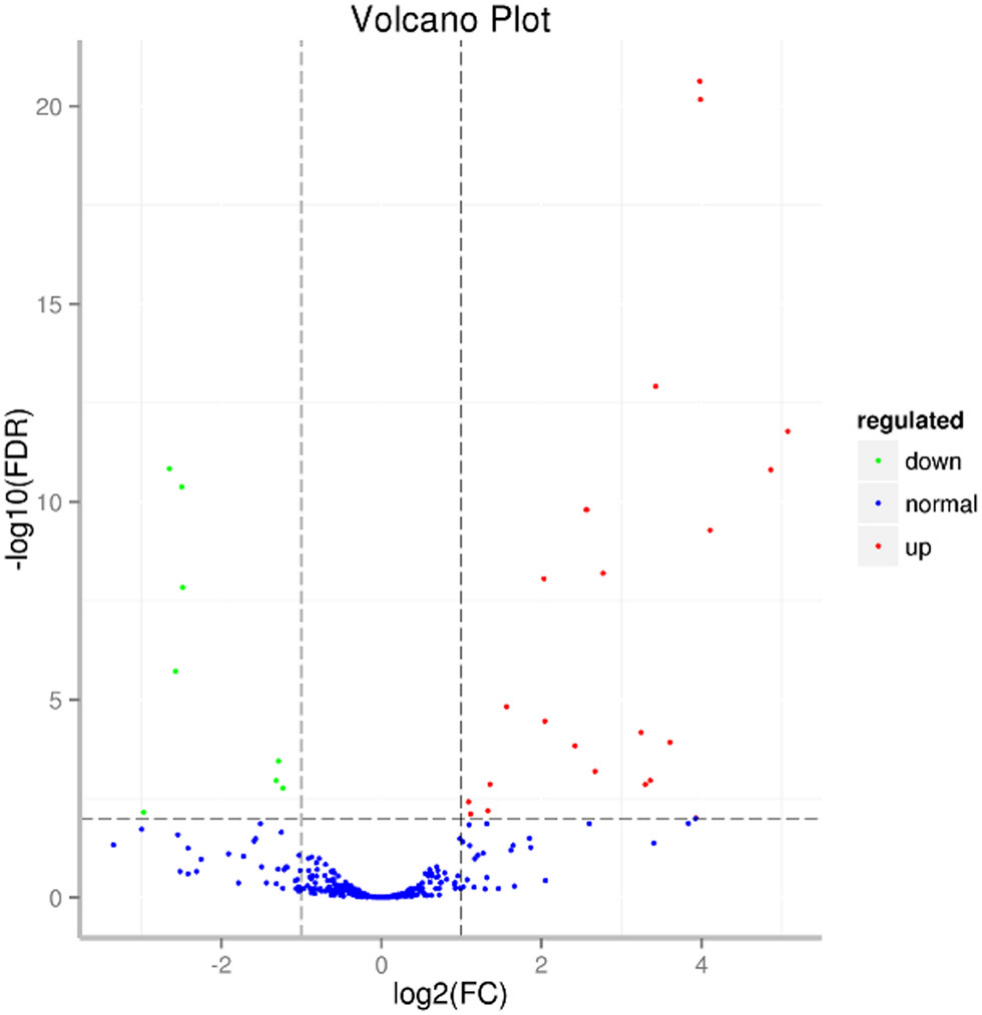
Differential expression analysis of miRNAs after drought treatment.

The drought-responsive DEmiRNAs analysis revealed that 44 zma-miRNAs exhibited differentially expressed patterns, including 32 known miRNAs and 12 novel miRNAs (Figure 6). Among these drought-responsive 44 DEmiRNAs, 35 DEmiRNAs were up-regulated while 9 DEmiRNAs were down-regulated (Figure 7).

### 3.4 Identification target genes of miRNAs by degradome analysis

**Figure 8:**
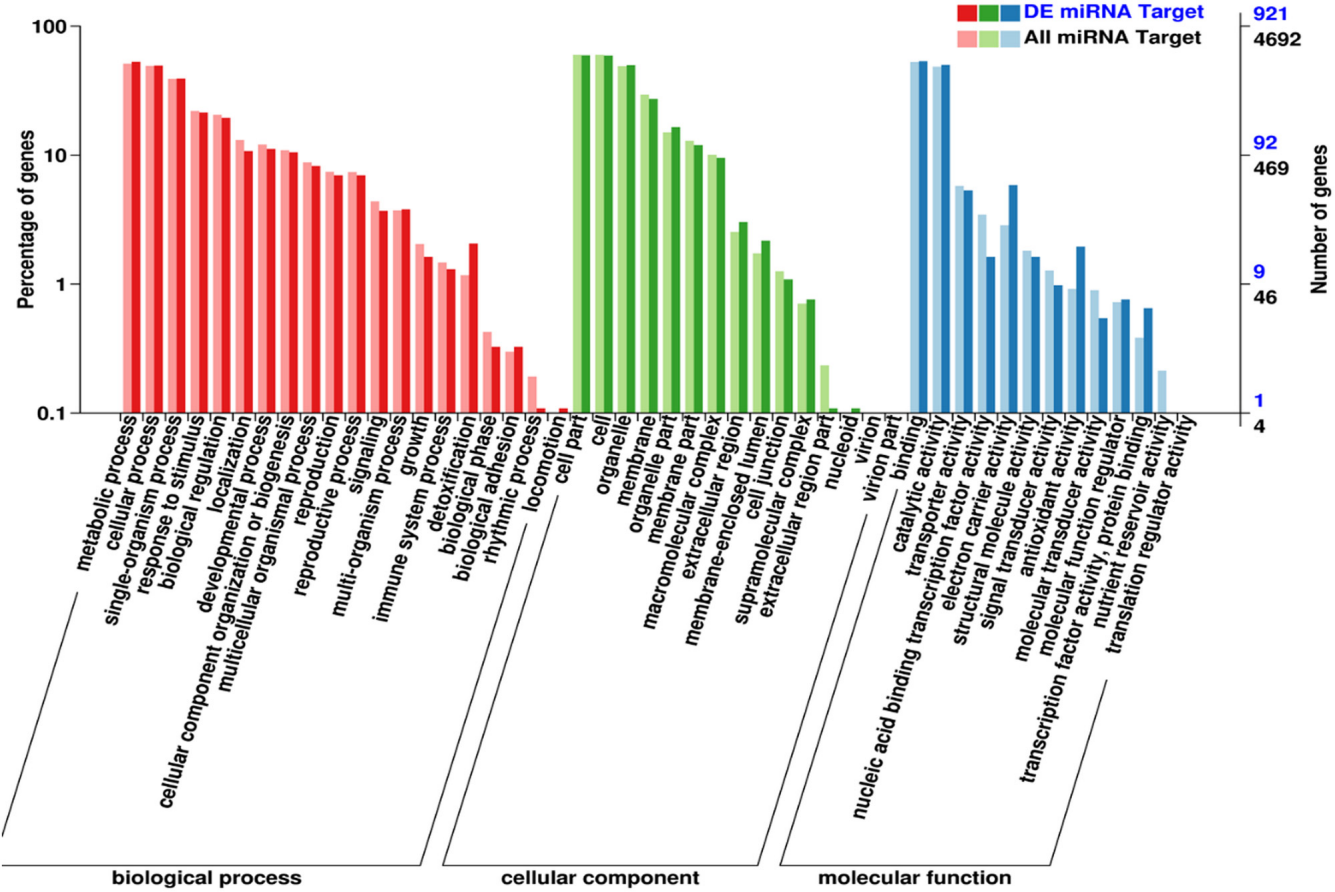
GO annotation of miRNA target genes.

Based on the degradome, 15,625,240 clean tags were obtained from the mixed sequencing pool. About 2,459,479 tags were mapped to the maize B73 genome, 6,276 unique tags were annotated as ncRNAs. Combined with the prediction software of target genes, about 6,340 genes were identified as the candidate targets of 445 miRNAs. These target genes were classified into 553 molecular functions, 234 cellular components, and 134 biological processes. Among the molecular function category, “binding” was the most abundant process with 239 unigenes (Figure 8). For biological process category, the most frequent process was “metabolic process,” followed by “cellular process.” Of the 15 processes of cellular component, “cell part” and “cell” were the most abundant.

**Figure 9:**
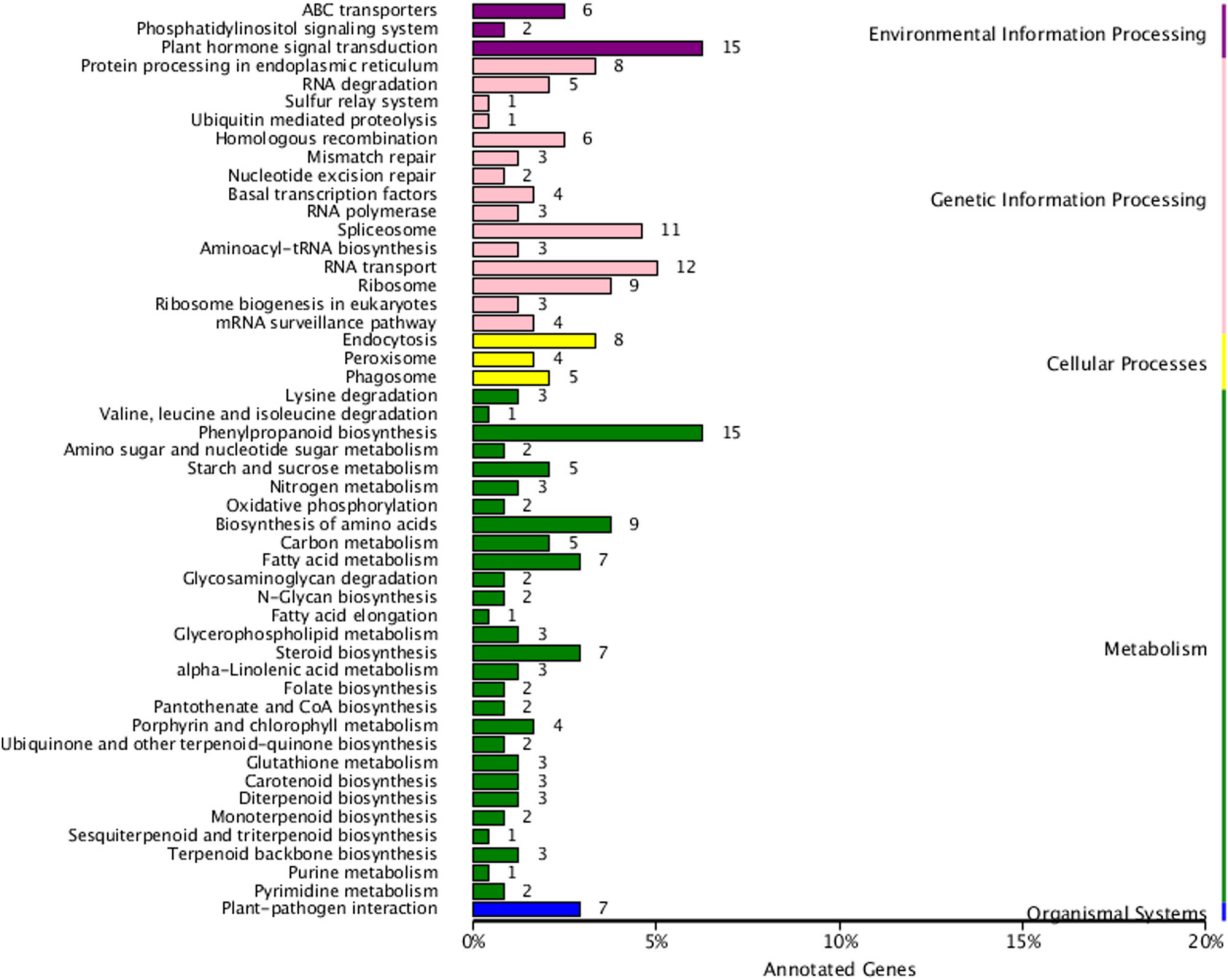
KEGG pathway analysis of miRNA target genes.

Subsequently, 220 predicted target genes were annotated into 50 different KEGG pathways, with most target genes enriched in the metabolism class. The “plant hormone signal transduction” pathway within the environmental information processing class and “phenylpropanoid biosynthesis” pathway within the metabolism class were both enriched in 15 unigenes, making them the most abundant pathways, meanwhile indicating the crucial roles of plant hormone and phenylpropanoid in the drought response of Zhengdan958 seedling roots. Among these different classes, “RNA transport” in genetic information processing, “endocytosis” in cellular processes, and “plant–pathogen interaction” in organismal systems were the most represented pathways (Figure 9).

### 3.5 Correlation analysis of the expression profiles of the DEmiRNAs and their target genes

**Figure 10:**
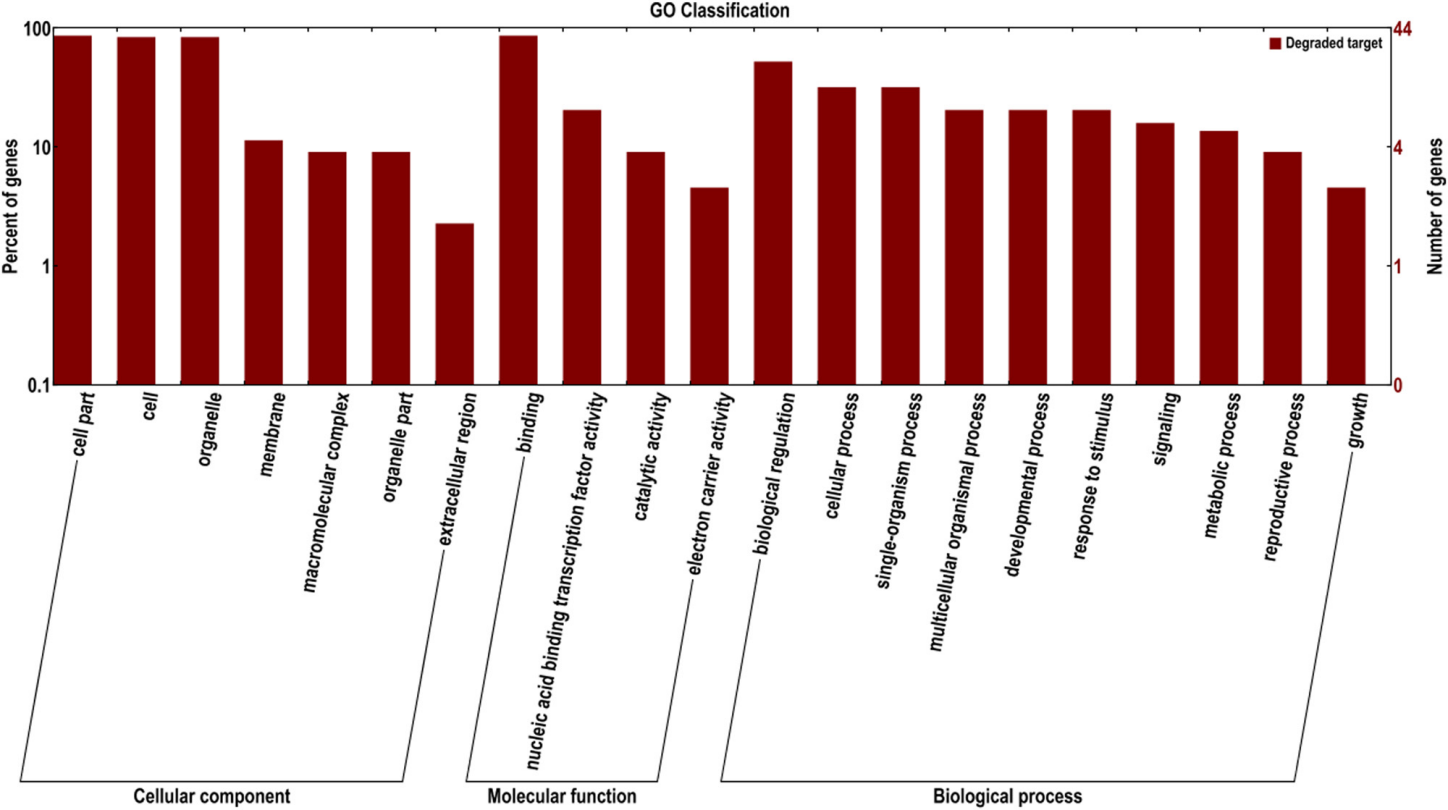
GO categories of DEmiRNA-target mRNAs.

A combined analysis of transciptome, sRNAome, and degradome was performed to identify the expression profiles of drought-responsive DEmiRNAs and their target genes. Of the 44 DEmiRNAs, 48 different mRNAs were predicted as the target genes of 25 DEmiRNAs, forming 281 DEmiRNA–mRNA pairs and these target genes were annotated into 21 different GO terms (Figure 10).

Of the 281 DEmiRNA–mRNA pairs, five pairs displayed the reversed regulation patterns when comparing DS with CK data (Table 1). For example, zma-miR528b-5p, zma-miR528a-5p, zma-miR408b-3p, zma-miR408a, and zma-miR164e-5p were up-regulated in response to drought stress, while their targets *Zm00001d021850*, *Zm00001d020794*, and *Zm00001d003414* were down-regulated correspondingly under drought stress.

**Table 1:** Reversed expression patterns of five DEmiRNA–mRNA pairs

DEmiRNA name	Regulation	Gene ID	Gene annotation	Regulation
zma-miR528b-5p	Up	*Zm00001d021850*	Cupredoxin superfamily protein	Down
zma-miR528a-5p	Up	*Zm00001d021850*		Down
zma-miR408b-3p	Up	*Zm00001d020794*	Blue copper protein	Down
zma-miR408a	Up	*Zm00001d020794*		Down
zma-miR164e-5p	Up	*Zm00001d003414*	NAC domain protein NAC5	Down

### 3.6 qRT-PCR validation of the reversed expression profile of five DEmiRNA–mRNA pairs

**Figure 11:**
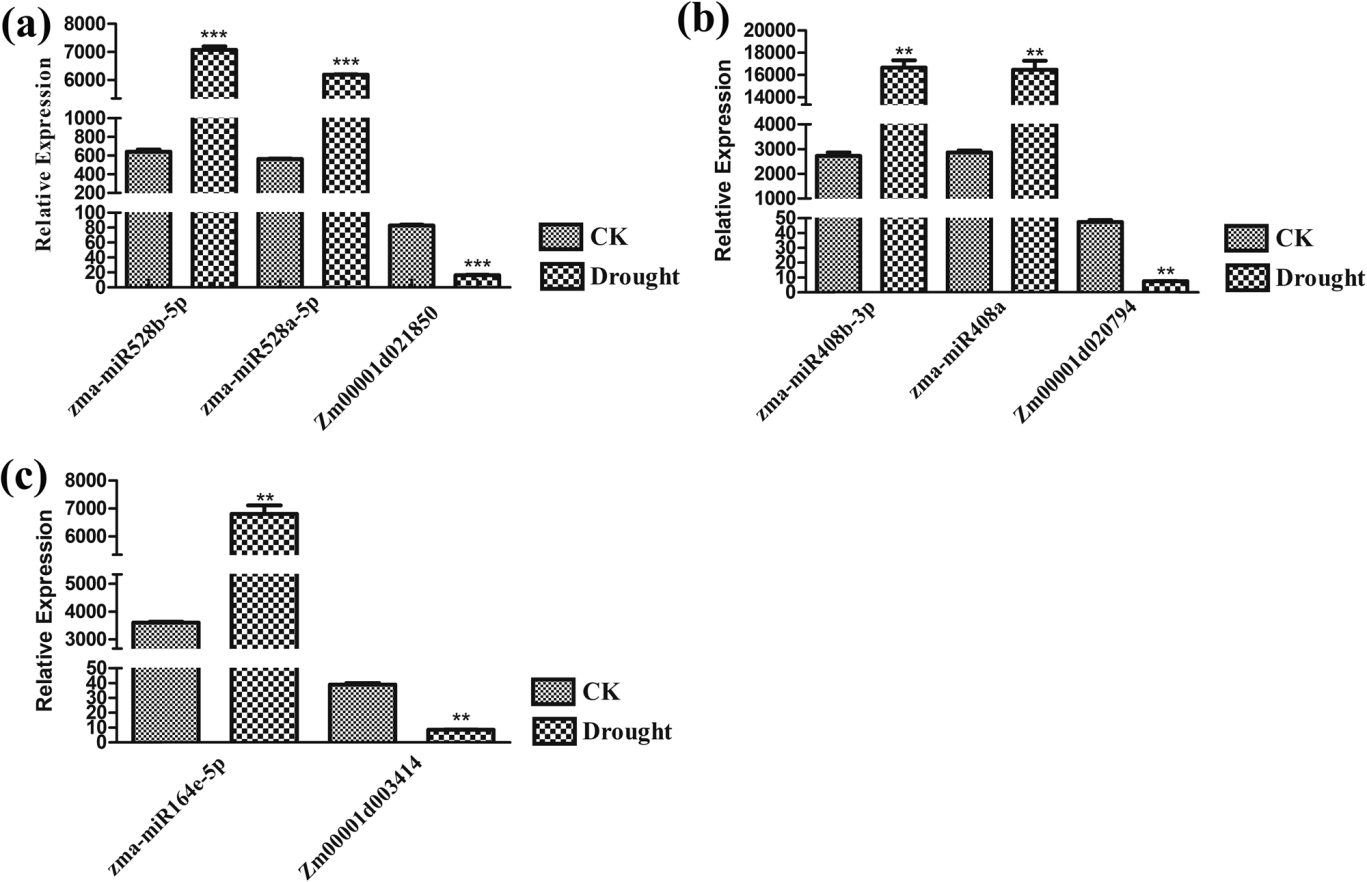
Comparison of the expression levels of five DEmiRNA–mRNA pairs by qRT-PCR. (a) Expression levels of zma-miR528b-5p, zma-miR528a-5p, and their target *Zm00001d021850*. (b) Expression levels of zma-miR408b-3p, zma-miR408a, and their target *Zm00001d020794*. (c) Expression levels of zma-miR164e-5p and target *Zm00001d003414*.

A qRT-PCR analysis was conducted to verify the reversed expression profile of five DEmiRNA–mRNA pairs associated with drought-response. The results showed that the expression trends of five DEmiRNA–mRNA pairs were similar to those obtained by multi-omics analysis (Figure 11). The expression levels of five DEmiRNAs and their target genes were confirmed to be negatively correlated, suggestting that the transcription of target mRNAs may be repressed by their corresponding miRNA.

### 3.7 Tissue-specific expression profiles of three DEmiRNA target genes

**Figure 12:**
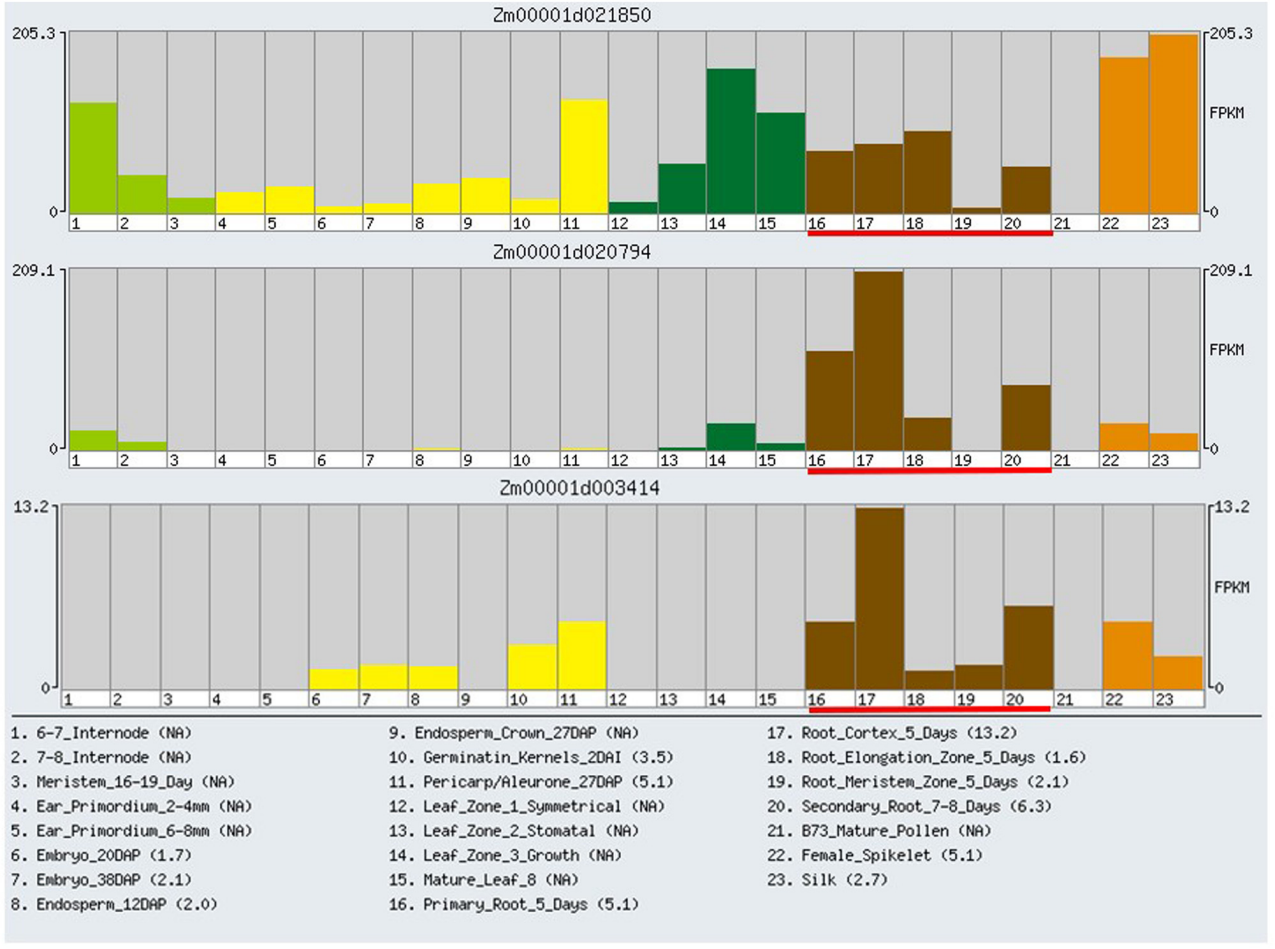
Tissue-specific expression profiles of three zma-miRNA target genes. *X*-axis shows the different tissues, *Y*-axis marked out the relative expression amount.

Based on the published transcriptome sequencing data from different tissues at different developmental stages of maize, the expression profiles of three DEmiRNA target genes in 23 tissues were analyzed (Figure 12). Our results showed that the transcriptional abundance of three DEmiRNA target genes were highly variable in different tissues, suggesting that they had multiple functions in maize growth and development. All the three DEmiRNA target genes showed a tissue-specific expression in the maize roots, indicating that these genes might participate in the root-specific stress response of Zhengdan958 seedlings.

### 3.8 Network of zma-miR528, zma-miR408, and zma-miR164 target genes involved in drought-responsive regulation

**Figure 13:**
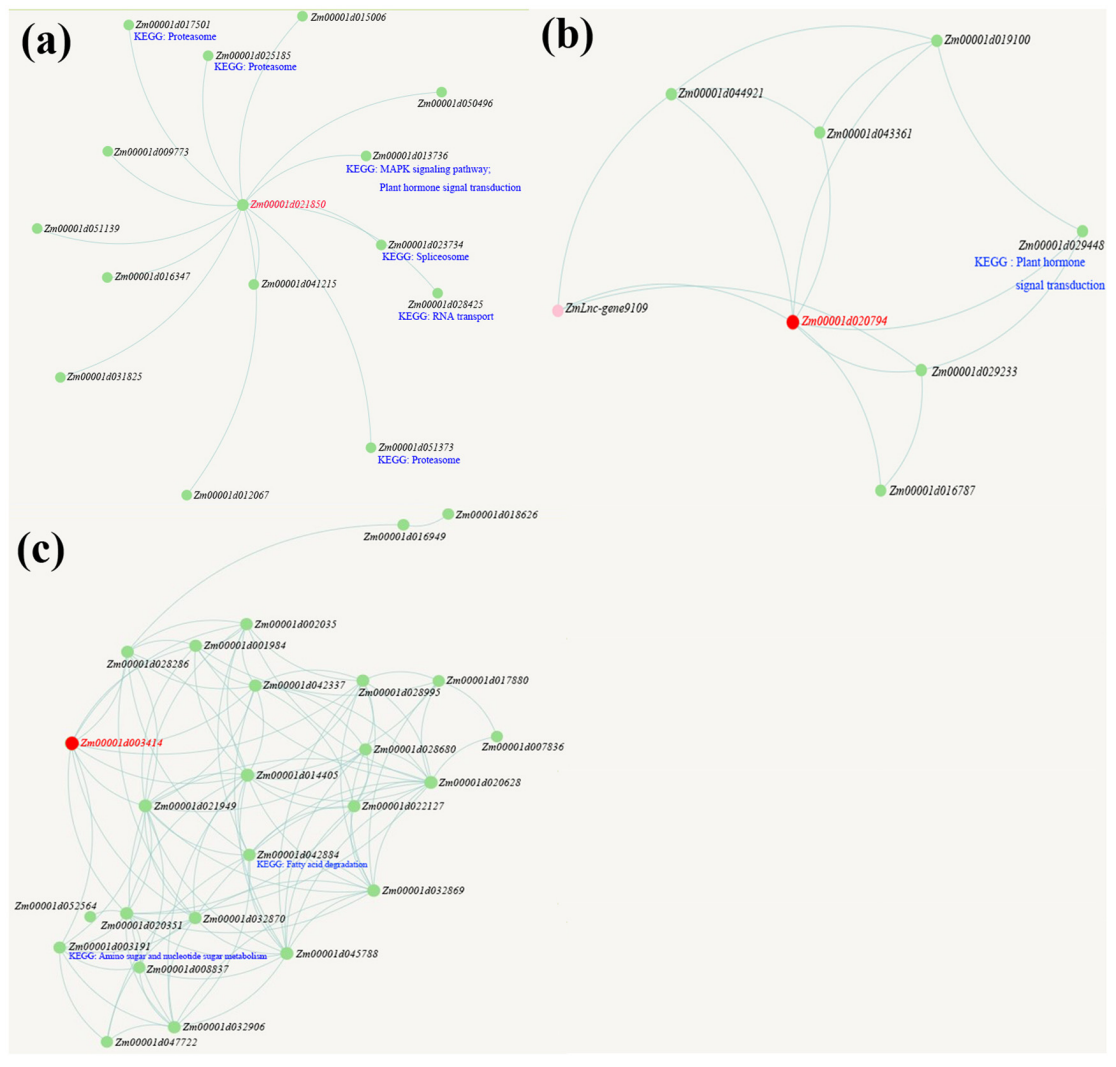
Co-expression subnetwork of *Zm00001d021850* (a), *Zm00001d020794* (b), *Zm00001d003414* (c), and their associated KEGG pathways.

The regulatory network and functional annotation of zma-miR528, zma-miR408, and zma-miR164 target genes and their interacting genes were deeply explored to better understand the mechanism of root-specific drought response in Zhengdan958 seedlings (Figure 13). Based on the databases of MaizeNetome, ATTED-II, and KEGG pathway, we found that *Zm00001d028425*, *Zm00001d025185*, *Zm00001d023734*, *Zm00001d013736*, *Zm00001d017501*, and *Zm00001d051373* display as the interacting gene of *Zm00001d021850*, which function in the KEGG pathways of RNA transport, peroxisome biogenesis, spliceosome, MAPK signaling pathway-plant, and plant hormone signal transduction. The *Zm00001d029448* displayed as the interacting gene of *Zm00001d020794*, which function in the KEGG pathway of plant hormone signal transduction. The *Zm00001d003191* and *Zm00001d042884* displayed as the interacting gene of *Zm00001d003414*, which function in the KEGG pathways of amino sugar and nucleotide sugar metabolism as well as fatty acid degradation.

## 4 Discussion

In this study, we used three high-throughput sequencing methods to study the probable drought-responsive mechanism of Zhengdan958 seedling roots. Transcriptome library was applied as a reference for the following sRNAome and degradome analysis, the integration of multi-omics helps to identify the drought-responsive miRNA–mRNA modules in Zhengdan958 seeding roots.

Based on the transcriptome sequencing, we obtained 43,284 genes including 3,960 new genes from six cDNA libraries. Through sRNAome analysis, 486 miRNAs belonging to 69 miRNA families were identified, 259 of which were novel miRNAs and 44 miRNAs displayed significantly different expression profiles between DS and CK. Some conserved miRNAs in responding to drought stress have also been detected, such as miR156, miR159, miR162, miR171, miR396, miR398, and miR408, indicating the important drought-responsive roles of these conserved miRNAs [26–31]. Twelve DEmiRNAs were identified as novel miRNAs, among which the expression of novel_miR_130, novel_miR_157, novel_miR_162, novel_miR_192, novel_miR_204, novel_miR_221, novel_miR_226, novel_miR_258, novel_miR_58, and novel_miR_76 were up-regulated, while novel_miR_227 and novel_miR_74 were down-regulated under drought stress.

Among these 44 DEmiRNAs, zma-miR408a, zma-miR408b-3p, and zma-miR408b-5p of the miR408 family were all up-regulated under drought stress, which displayed a conserved drought-responsive up-regulation with that in *M. truncatula* [32]. However, other studies indicated that miR408 displayed a decreasing expression owing to water deprivation in *Pisum sativum* and *M*edicago *ruthenica* [2,31]. These differences might be caused by the drought extent and duration of different species, along with the sensitivity of some miRNAs to subtle differences during plant growth [32]. However, different members from the same miRNA family even might function in a species-specific manner [33]. Some zam-miRNA members from the miR156 and miR157 family were observed to display differently expressed patterns with other crops in the drought-response of Zhengdan958 seedling roots. Similar results have also been observed in *O. sativa*, nine conserved miRNAs (osa-miR156, osa-miR168, osa-miR170, osa-miR171, osa-miR172, osa-miR319, osa-miR396, osa-miR397, and osa-miR408) showed opposite expression patterns with that observed in the drought-stressed *Arabidopsis* [34].

Generally, a single mRNA could be regulated by several miRNAs, meanwhile a single miRNA could target several mRNAs [35,36]. Three members (zma-miR169i-3p, zma-miR169j-3p, and zma-miR169k-3p) of the miR169 family enriched in the most abundant target genes among all DEmiRNAs, of which both had 479 target genes in the CK and DS library. In *A. thaliana*, the miR169 family was also the largest miRNA family. Asefpour Vakilian [10] found that the miR169 displayed the highest drought-responsive levels in *A. thaliana* through the feature selection algorithm. Expression analysis of ath-miR169 precursors indicated that ath-miR169a was substantially down-regulated under drought stress, which mainly regulated the gene expression of NFYA5 at the mRNA level [37,38]. KEGG analysis of 193 zma-miR169 target genes was annotated into 137 different pathways, such as plant hormone signal transduction (K16189), MAPK signaling pathway-plant (K13414), homologous recombination (K10869), and ribosome biogenesis in eukaryotes (K07179), revealing the extensive regulatory roles of zma-miR169 target genes in maize expect for drought-response.

Based on the high-throughput sequencing technology and bioinformatics analysis, degradome sequencing has effectively avoided the false positive results and been a more suitable method for the target gene identification of plant miRNAs [39]. By means of the correlation analysis of transcriptome, sRNAome, and degradome, the association relationships of DEmiRNAs-target genes were obtained. Among these DEmiRNA–mRNA pairs, zma-miR528a-5p, zma-miR528b-5p, zma-miR408a, zma-miR408b-3p, and zma-miR164e-5p and their target genes displayed negative regulatory relationships in the drought response of Zhengdan958 seedling roots, which were also verified by qRT-PCR. In *Agropyron mongolicum*, drought-responsive bdi-miR408-5p-*CCX1* and bdi-miR528-p3-*HOX24* mediated the brassinosteroid signal pathway, transporting and exchanging sodium and potassium ions and regulating the oxidation–reduction process, hydrolase activity, plant response to water deprivation, ABA, and the ABA-activated signaling pathway to regulate drought stress [40]. By regulating the development of secondary cell wall, zma-miR408-*LAC9* negatively regulated the salt tolerance in maize; meanwhile, zma-miR164-*NAC* modulated the stress response of the tolerant maize line in an ABA-dependent manner [41,42]. In this study, the drought-responsive zma-miR164 also down-regulated its target *Zm00001d003414* (*NAC5*) in Zhengdan958 seedling roots under drought stress. During the somatic embryogenesis process of maize, zma-miR528 regulated multi-target mRNAs (e.g., *PLC1*, *LAC3/5*, and *SOD1a*) by promoting their degradation, translation inhibition, or both [43]. KEGG enrichment analysis showed that the interacting genes of zma-miRNA528 and zma-miRNA408 target genes were mainly involved in the pathways of peroxisome biogenesis (PEX19 and SOD), plant hormone signal transduction (SnRK2 and JAZs), and so on. Our results revealed that the drought-responsive miRNA–mRNA modules not only regulated the mRNA levels of the miRNA-target genes, but also participated in the regulatory and interaction network of miRNA-target genes in Zhengdan958 seedling roots. Although the complex miRNA–mRNA regulatory networks still needed to be elucidated, our findings provided valuable information for further functional characterization of drought-responsive miRNA–mRNA modules in maize roots. More importantly, this study will also serve as a foundation for functional research of miRNA–mRNA regulatory modules in Poaceae species.
